# Effects of zearalenone and α-Zearalenol in comparison with Raloxifene on T47D cells

**DOI:** 10.1080/15376510802455347

**Published:** 2009-06-30

**Authors:** Roya Khosrokhavar, Nahid Rahimifard, Shahram Shoeibi, Morteza Pirali Hamedani, Mir-Jamal Hosseini

**Affiliations:** 1 Food and Drug Laboratory Research Center (FDLRC), Ministry of Health (MOH), Tehran, Iran; 2 Department of Medicinal Chemistry, Faculty of Pharmacy, Tehran University of Medical Sciences (TUMS), Tehran, Iran; 3 Department of Pharmacology, Faculty of Medicine, Tehran University of Medical Sciences, Tehran, Iran

**Keywords:** Zearalenone, α-Zearalenol, Raloxifene, Estrogenic activity, Breast cancer

## Abstract

Zearalenone (Zen) is a mycotoxin with estrogenic effect which contaminates cereals. In cell culture, Zen and its metabolite, α-Zearalenol (α-Zel), stimulate breast cancer cells growth. Today hormone-dependent cancers are important because of high incidence and death rate. Previous studies showed that Zen and α-Zel have an effect on hormone-dependent cancers. This study explains the effects of the mentioned compounds in comparison with Raloxifene as an anti-estrogen. Cell culture technique was used with MDA-MB-231 and T47D cells for evaluation of compounds. MDA-MB-231 cells were used as negative control and also for proving that treatment compounds merely affect, due to their proliferation activity in the applied doses. According to the Resazurine-based method, for toxicity assay, none of the test compounds have an effect on MDA-MB-231 cells but do effect the growth of T47D cells. Zen and α-Zel at low concentrations (10–8–10–9 M) stimulated T47D cell growth and Raloxifene strongly inhibited cell growth induced by Zen and α-Zel. There is a noticeable result in controlling diet of hormonal carcinogenic compounds and applying novel anti-estrogens for prevention and treatment of hormone-dependent cancers.

## Introduction

Estrogen mimics (xenoestrogens) are a group of environmental contaminants which cause reproductive dysfunctions in wildlife and interfere with cancer diseases in humans. Natural or synthetic estrogenic compounds can induce cell proliferation, hypertrophy of female secondary sex organs and the synthesis of cell type-specific proteins (Hertz 1985). Dietary estrogens are either produced by plants (phytoestrogens) or by fungi (myco-estrogens) that infect plants or their products (Kurzer and Xu 1997).

The human diet contains several non-estroidal estrogenic compounds, which are structurally similar to natural or synthetic estrogens or anti-estrogens. Zearalenone (Zen) is a myco-estrogen with non-estrogenic chemical structure (resorcylic acid lactones) which is produced by several species of *Fusarium* fungi and widely contaminates agricultural products (Bennet and Shotwel 1979; Sutton et al. 1980; Trenholm et al. 1985). Zen exists in almost every agricultural product including cereals, mixed feeds, and rice, but corn is the most frequently contaminated commodity (Tanak et al. 1988; Gareis et al. 1989; Veldman et al. 1992). After consumption, Zen can be metabolized in the body by reduction of the keto-group at C-6′ and convertion to two major stereo isomers metabolites, α- and β-Zearalenol (α- and β-Zel) (Kiessling and Pattersson 1978; Mirocha et al. 1981; Kuiper-Goodman et al. 1987). The more potent estrogenic active metabolite of Zen is α-Zel, which is also naturally produced by fungi (Richardson et al. 1985; Erasmuson et al. 1994).

The effect of these myco-estrogens in animals is *Estrogenic Syndrome* with clinical hyperestrogenism manifestations in those fed contaminated grains, and causes infertility as a major problem in animal husbandry, especially in swine (Chang et al. 1979; Etienne and Jemmali 1982). Also, some derivatives of zearalenone like Zeranol (Ralgro), a synthetic product, have androgenic activity and are used as growth promotors in animals (Baldwin et al. 1983) and humans, because of the estrogenic properties, can interact to hormone receptors and play an important role in hormone-dependent diseases like breast (Martin et al. 1978; Makela and Davis 1994; Dees et al. 1997; Zava et al. 1997) and endometrium cancer (Tomaszewski et al. 1998; Withanage et al. 2001).

Recently, environmental scientists have paid attention to the relationship between dietary estrogens and hormone-dependent cancer (Barnes 1998; Clarke et al. 2001), especially breast cancer, which is the most common cause of death in women worldwide (Key et al. 2001; Lacey et al. 2002). Although their relative contribution to the genesis of breast cancer in humans appears to be controversial (Safe 1997), xenoestrogenic compounds can add to the estrogenic burden and therefore could be a potential source of estrogenic exposure in women, especially in prepubertal and postmenopausal years. Some studies have shown that Zen stimulates the growth of MCF-7 breast cancer cell lines in vitro (Makela and Davis 1994; Zava et al. 1997), and enlarge mammary tumors in mice (Schoental 1974), reflecting its estrogen receptor-agonist properties.

In present study, we examined the effect of Zen and α-Zel at different concentrations on T47D, estrogen receptor-positive, and MDA-MB-231, estrogen receptor-negative breast cancer cell lines as negative control cells, for in vitro model determination of these myco-estrogens and 17-β-Estradiol (E2) as positive control agent. We also investigated the preventive effect of Raloxifene, new SERMs (Selective Estrogen Receptor Modulators) with mixed agonist/antagonist properties, on estrogen receptor, and identified the potential reduction in the risk of breast cancer (Barrett-connor 2001; Kim et al. 2002). The effects of Raloxifene on the above mentioned cell lines alone and in combination with test myco-estrogens and E_2_ have been studied to show the anti-estrogenic effect on T47D cells.

## Materials and methods

### Chemicals

Zearalenone, α-Zearalenol, 17-β-Estradiol, and Raloxifene were purchased from Sigma (Steinem, Germany). The stock solutions of estrogenic compounds were prepared in absolute ethanol. The final concentration of solvent was 0.5% (v/v). The stock solutions were stored at 2–8°C. Serial dilutions for all compounds were made in culture medium. RPMI 1640, Dulbecco's Modified Eagle Medium or DMEM, and their phenolred-free, Fetal Bovine Serum (FBS), L-Glutamine, Trypsin-EDTA, and Penicillin-Streptomycin were purchased from Gibco BRL (Grand Island, NY), and Charcoal-Dextran Treated FBS purchases from Hyclone (Logan, Utah).

### Cell lines and culture

Breast cancer human cell lines, T47D, passage number 18–25 (estrogen receptor-positive, ER+), and MDA-MB-231, passage number 44-51 (estrogen receptor-negative, ER−) were obtained from American Type Culture Collection (Manassas, Virginia). T47D cells were maintained in RPMI 1640 with added 5% FBS, 1% L-Glutamine, and Penicillin-Streptomycin, and then cells were incubated at 37°C in a 5% concentration of CO_2_. MDA-MB-231 were maintained in DMEM with added 10% FBS, 1% L-Glutamine, and Penicillin-Streptomycin at 37°C in a 5% concentration of CO_2_. Two days before experiment, the media of cells were removed and phenol red-free medium with Charcoal Dextran Treated FBS was added to cells.

### Experiments

Cells were harvested by Trypsin-EDTA and resuspended in fresh medium and seeded in 96-well culture plates at 4000 cells/well. After 1 day incubation in order to cells attaching, different concentrations of test compounds were prepared by serial dilution (1:10) and each concentration added to wells as quadrupled from 10^−4^M to 10^−14^M. In each plate, there were control wells (cells without test compounds) and blank wells (the medium only). On day 3 the medium was removed and fresh medium with these compounds were added to cells. All compounds were added to cells alone to observe the effects and then Zen, α-Zel, E2 in combination with Raloxifene, added to the cells. After 7 days the plates were prepared for viability assay.

### Viability Assay

In order to determinate cell viability, the Resazurine-based method was used. Briefly, the method is designated for spectrophotometrically or fluorometricaily determining cell numbers as a function of metabolic activity using the dye Resazurine. Bio-reduction of the dye by viable cells reduces the amount of its oxidized form (blue) and concomitantly increases the amount of its fluorescent intermediate (red), indicating the degree of viability caused by the test compounds. After the exposure time of compounds, the media of plates were removed and 10% (v/v) Resazurine in phenol red-free medium with Charcoal Dextran treated with FBS was added to cells in plates and placed in the incubator. Between 2–4 h of exposure to the dye, the plates were removed from the incubator and the viability was measured fluorometrically while monitoring the increase in fluorescence at a wavelength 590 nm using an excitation wavelength of 560 nm by multi-plate reader. The plate reader was a dual-scanning spectrofluorometer, SPECTR Amax GEMINI XS made by Molecular Devices Co. (Sunnyvale, CA).

### Statistical analysis

Data are presented as mean ± SD. Significance of results was assayed by using ANOVA, followed by Dennett's Comparison of Treatment against Control with *p* < 0.001 evaluated as statistically significant.

## Result

**Figure 1:**
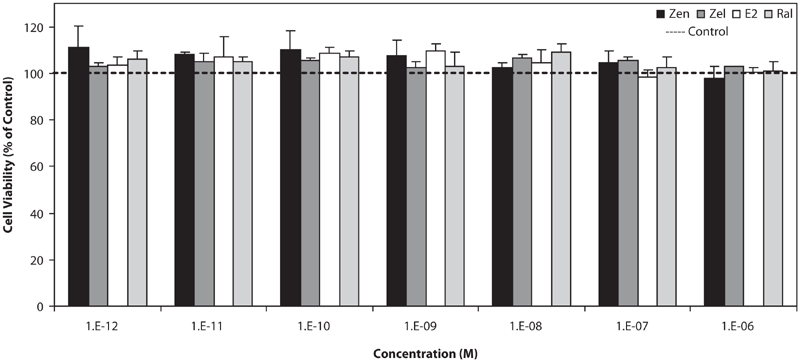
Effects of Zen, α-Zel, E_2_, and Raloxifene on growth of MDA-MB-231 cells. Cells were seeded in 96-wells (4 × 10^3^/well) and after 7 days of exposure to different concentrations of test compounds, cell viability was determined by Resazurine-based method. Data are presented as mean ± SD (n = 4).

MDA-MB-231 cells (estrogen receptor-negative breast cancer line) were used as negative control for testing the estrogenic effect of tested compounds. As shown in Figure 1, none of the estrogenic test compounds had an effect on growth of this cell line in the used concentrations (10^−6^–10^−12^M), neither stimulation nor suppression.

**Figure 2:**
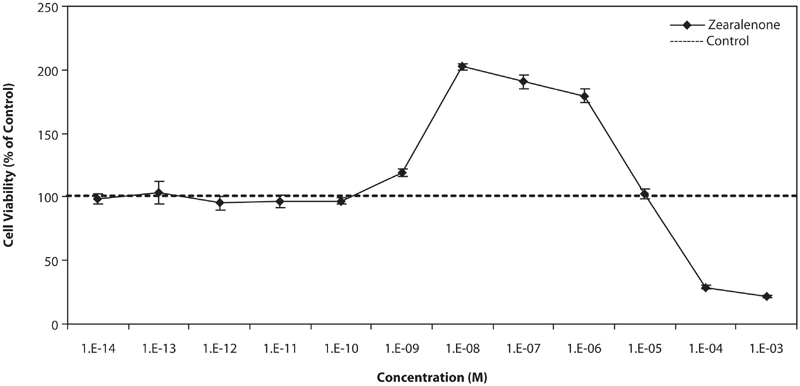
Effects of different concentration of Zen on growth of T47D cells. Cells were seeded in 96-wells (4 × 10^3^/well) and after 7 days of exposure to different concentrations of test compounds, cell viability was determined by Resazurine-based method. Data are presented as mean ± SD (n = 4).

Figure 2 shows the effect of different concentrations of Zen on T47D cells. At low concentrations, below 10^−9^M, there were no effects on growth of cells, at concentrations between 10^−9^ M and 10^−6^ M, the stimulatory effect was observed and the most was at 10^−8^ M, about 2-fold stimulation of the growth in comparison with control cells. At high concentrations, above 10^−6^ M, the growth was suppressed and it seems that the toxic effect of Zen on cells appears in these concentrations.

**Figure 3:**
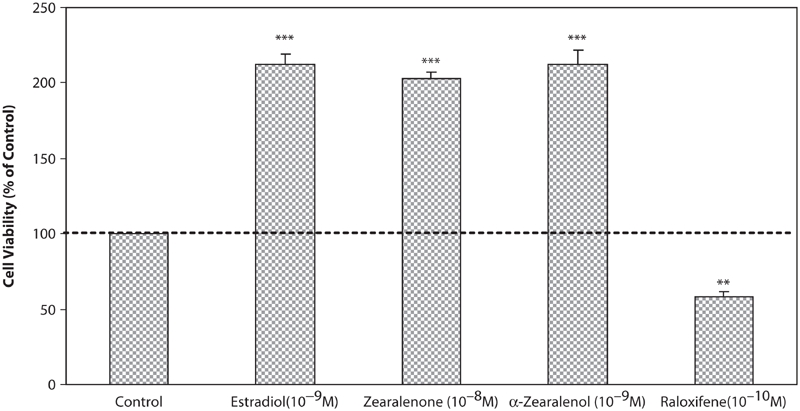
Effects of Zen, α-Zel, E2, and Raloxifene on growth of T47D cells. Cells were seeded in 96-wells (4 × 10^3^/well) and after 7 days of exposure to different concentrations of test compounds, cell viability was determined by Resazurine-based method. Data are presented as mean ± SD (n = 4). ** p < 0.01, ***p < 0.001.

In Figure 3 the effect of test compounds on T47D cells were shown. E2, a potent ligand for estrogenic receptors, at concentration of 10^−8^ M, has shown the most stimulatory effect on the growth of cells as about 2-fold in comparison with control cells. Also, Zen and α-Zel have stimulated the growth of cells at low concentrations (Zen 10^−8^ M and α-Zel 10^−9^ M), which was comparable with E2. Raloxifene, a novel SERMs, strongly inhibited the growth of cells at low concentration (10^−10^ M).

**Figure 4:**
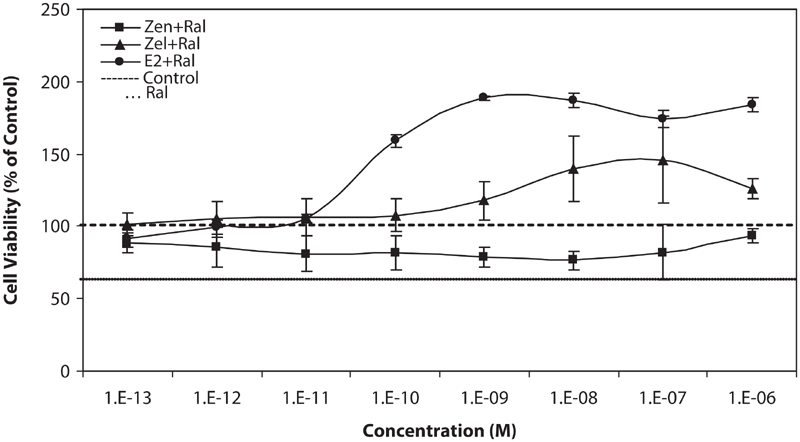
Effects of equivalent concentrations of Zen, α-Zel, and E_2_ with Raloxifene on growth of T47D cells. Cells were seeded in 96-wells (4 × 10^3^/well) and after 7 days of exposure to different concentrations of test compounds, cell viability was determined by Resazurine-based method. Data are presented as mean ± SD (n = 4).

We also examined the simultaneous effect of equivalent concentration of stimulating test compounds (Zen, α-Zel, and E2) against Raloxifene, which is shown in Figure 4. Raloxifene could suppress the stimulatory growth effect of Zen from 10^−12^ to 10^−7^ M concentrations in comparison with the results shown in Figure 2.

α-Zel, which is more potent than Zen, could stimulate the proliferation of T47D cells from 10^−9^ to 10^−6^ M concentrations, although Raloxifene was inhibited from this effect moderately.

E_2_, as the most potent ligand for estrogen receptor, had a stronger effect on cell proliferation from 10^−9^ M concentration and higher, the inhibitory effect of Raloxifene was weaker in comparison with other compounds.

## Discussion

Hormone-dependent cancers are one of the most important diseases because of their high incidence and death rate. Breast and endometrial cancers are examples of the mentioned diseases which mostly are estrogen-receptor dependent, thus chemicals with estrogenic properties may effect the promotion or prevention of this type of cancers.

Diet is one of the main factors in inducing, promoting, or preventing cancer. Foods include different components; some of them, like vitamins, minerals, and fibers, are useful in prevention and treatment of cancers, and some are harmful. Natural compounds like phyto- and myco-estrogens can interfere with hormonal receptors and affect hormone-dependent cancers.

In this investigation we studied the response of affected cells by compounds with estrogenic properties. We used two types of breast cancer cell lines: MDA-MB-231 (ER−) as negative control and T47D (ER+) in order to observe that test compounds affect via interfering with estrogen receptors. In comparison between results shown in Figure 1 and Figure 3, it is observed that test compounds interfere with receptors and resulted in this effect. Anyway the mentioned compounds may have other carcinogenic effects except proliferation of cells. The effect on MDA-MB-231 impresses that, in applied doses of test compounds, they did not have any effect on cellular level, but maybe in higher doses, they could have a cytotoxic effect, which is shown in Figure 2, at higher than 10^−5^ M concentrations. It could be also considered that, according to the aim of study, which is interaction between potent estrogens against novel SERMs (Raloxifene), test compounds did not have any molecular effect, which concluded to proliferation respond.

Figure 3 shows that Zen and α-Zel have strong effects on growth stimulation comparable with E2. The last results show that these mycotoxins can play an important role in promotion of estrogen-dependent cancer and cause proliferation on related cells. Zen, after ingestion by humans or animals, can affect cells directly, or be metabolized by organs and converted to α-Zel, which is more potent than the primary compound.

Raloxifene, a novel SERM, when applied against Zen, α-Zel, and E2 at equivalent concentrations, inhibits the increased proliferation stimulated by test compounds, which is shown in Figure 4. After further in vivo studies and clinical trials it could be considered as one of the helpful compound in prevention and treatment of hormone-dependent cancer against biological or synthetic chemical with estrogenic properties, and has protective effect on cells containing estrogen receptors.

It is suggested to study other compounds with estrogenic properties which have important roles in nutrition and environmental conditions of life. Also, because of the dose-dependent response of these compounds, some studies are focused on the dose–response effect and interaction with anti-estrogens together with anti-cancer agents like vitamins.

Raloxifene alone could significantly suppress normal growth of T47D cells by about 40% (*p* < 0.01). This matter could be suggested for further studies. Raloxifene effectively inhibited cell proliferation induced by Zen, so whereas Zen is one of the hazards in human diet, anti-estrogens could be effective in peoples who consume agricultural products more in their diet, especially corn products.

α-Zel has been moderately suppressed by Raloxifene, although the stimulatory effect of it was observed. α-Zel does not directly exist in the diet, it is produced by metabolization in the body and also could be a more hazardous compound.
